# President's address: Improving the quality of testing

**DOI:** 10.3352/jeehp.2011.8.1

**Published:** 2011-01-03

**Authors:** Kun Sang Kim

**Affiliations:** National Health Personnel Licensing Examination Board, Seoul, Korea.

The National Health Personnel Licensing Examination Board (NHPLEB) of the Republic of Korea completed the clinical skill test for the Medical Licensing Examination in 2009 and 2010 that was introduced for the first time in Asia. This was only possible due to the great amount of time devoted by the medical professors and staff of NHPLE. To improve the evaluation of what examinees can do instead of only what they know, a clinical skill test should also be administered in other health professional fields. Recently, the NHPLEB has dealt with 160,000 examinees including licensed practical nurses, caregivers, and certified health education specialists. The NHPLE will apply to the ISO 9001 certification process in 2011. Also, the work of the ad hoc team for computer-based testing will be extended in 2011. On December 14, 2010, during the meeting with the President of the Association for Medical Education in the Western Pacific Region (AMEWPR), Dr. Duck-Sun Ahn, it was agreed that the *Journal of Educational Evaluation for Health Professions* will be an official journal of the AMEWPR beginning in July 2011 after going through a scheduled process. Workshops for the capability on how to develop test item will be continued specific to each field so that the pool of examiners can be expanded. The year 2012 is the 20th anniversary of the NHPLEB. Thus preparations for the international conference to celebrate the 20th anniversary will begin in 2011. I appreciate all readers and the health personnel educators for their support of the NHPLEB in 2010. I will persevere in my effort to develop the National Health Personnel Licensing Examination in every way in 2011.

Happy New Year!

